# Fasting Induced Cytoplasmic *Fto* expression in Some Neurons of Rat Hypothalamus

**DOI:** 10.1371/journal.pone.0063694

**Published:** 2013-05-06

**Authors:** Predrag Vujovic, Stefan Stamenkovic, Nebojsa Jasnic, Iva Lakic, Sinisa F. Djurasevic, Gordana Cvijic, Jelena Djordjevic

**Affiliations:** 1 Institute of Physiology and Biochemistry, Faculty of Biology, University of Belgrade, Belgrade, Serbia; 2 Center for Laser Microscopy, Faculty of Biology, University of Belgrade, Belgrade, Serbia; Hosptial Infantil Universitario Niño Jesús, CIBEROBN, Spain

## Abstract

Fat mass and obesity associated protein (*Fto*) is a nucleic acid demethylase, with a preference for thymine or uracil, according to the recent structural data. This fact suggests that methylated single-stranded RNA, rather than DNA, may be the primary *Fto* substrate. *Fto* is abundantly expressed in all hypothalamic sites governing feeding behavior. Considering that selective modulation of *Fto* levels in the hypothalamus can influence food intake, we set out to investigate the effect of 48 h fasting on the *Fto* expression in lateral hypothalamic area, paraventricular, ventromedial and arcuate nucleus, the regulatory centres of energy homeostasis. We have demonstrated that 48 h fasting causes not only an increase in the overall hypothalamic levels of both *Fto* mRNA and protein, but also alters *Fto* intracellular distribution. This switch happens in some neurons of paraventricular and ventromedial nucleus, as well as lateral hypothalamic area, resulting in the majority of the enzyme being localized outside the cell nuclei. Interestingly, the change in the *Fto* intracellular localization was not observed in neurons of arcuate nucleus, suggesting that fasting did not universally affect *Fto* in all of the hypothalmic sites involved in energy homeostasis regulation. Both *Fto* mRNA and catechol-O-methyltransferaze mRNA were upregulated in the identical time-dependent manner in fasting animals. This fact, combined with the knowledge of the *Fto* substrate preference, may provide further insight into monoamine metabolism in the state of disturbed energy homeostasis.

## Introduction

Fat mass and obesity associated protein (*Fto*) is an AlkB-like 2-oxoglutarate – dependent nucleic acid demethylase, which exhibits substrate specificity for 3-methylthymidine and 3-methyluracil in single-stranded DNA and RNA [1]. However, recent structural data [2] show that specific hydrogen bond interactions account for the preference of *Fto* for thymine or uracil over adenine, cytosine or guanine, suggesting that methylated single-stranded RNA, rather than DNA, may be the primary *Fto* substrate. Sequence analysis also predicted that human *Fto* and its vertebrate homologs are globular proteins that carry a nuclear localization signal (NLS) and are unlikely to be targeted to membranes or organelles [3].

The studies of both rodent and human *Fto* mRNA expression have shown that this protein is expressed in many tissues (e.g. pituitary, heart, kidney, white and brown adipose tissue), [4], [5], with concentrations especially high in the hypothalamic sites that govern feeding behavior, such as arcuate (ARC), paraventricular (PVN), dorsomedial and ventromedial (VMN) nuclei [6].

Although the connection between single nucleotide polymorphisms in the *Fto* gene and body mass index was established long ago [7], [8], downstream effects of changes in *Fto* expression remain unexplored. Animal experiments indicate a relationship between *Fto* levels and energy metabolism and food intake, impacting body weight. For instance, it has been reported that *Fto* expression was significantly increased in the hypothalamus of food-deprived and food-restricted rats [6].

Considering that selective modulation of *Fto* levels in the hypothalamus influences food intake [9], our goal was to determine the effect of fasting on *Fto* expression in lateral hypothalamic area (LHA), PVN, VMN and ARC, all of which are the regulatory centres of energy homeostasis.

## Materials and Methods

The experiments were conducted on adult male *Wistar* rats, weighing (250±20) g, bred in the vivarium of the Belgrade University Faculty of Biology. Two rats were housed per cage under the controlled temperature conditions (21±1)°C and lighting (12 h light –12 h of darkness). The food was removed at the onset of the dark phase (6.00 p.m) and the animals remained food deprived for 48 h. The group subjected to fasting was sacrificed simultaneously with the *ad libitum* fed control group,. All the animals had free access to tap water. The experiment was performed according to the rules for animal care proposed by the Serbian Laboratory Animal Science Association, a member of the Federation of European Laboratory Animal Science, and approved by the Ethics Committee of the Faculty of Biology, University of Belgrade.

Animals were decapitated without anaesthesia using a guillotine (Harvard-Apparatus, Holliston, MA). The brains were quickly excised, hypothalami were removed and then frozen at –80°C until further use for RT-qPCR, Western Blotting or Immunofluorescence.

### Tissue sample preparation for Western blotting

After decapitation, rat hypothalami were homogenized on ice with an Ultra-turrax homogenizer in buffer (pH 7.4) containing (in mM): 150 NaCl, 10 Tris, 1 EDTA; 10% Glycerol, 1% Triton X-100, Protease inhibitor cocktail with additional 2 mM PMSF, and 2 mM sodium orthovanadate. Homogenates were centrifuged at 600×g for 20 min at 4°C, and the supernatants were ultracentrifuged for 60 min at 100,000×g. Protein concentration was determined by the BCA method [10].

### SDS-PAGE and Western blot

Protein lysates (1 mg per line) were separated by 12 % SDS polyacrilamide gels and transferred onto polyvinylidene fluoride membranes. After Ponceau S staining and destaining, the membranes were blocked for 1 h in 5% nonfat dry powder milk (Santa Cruz) in Tris-buffered saline containing 0,1% Tween 20 (TBST) and probed with goat polyclonal antibody directly against *Fto* (1∶1000 dilution, ab77547), overnight at 4°C on a shaker. After washing, membranes were incubated with rabbit polyclonal secondary antibody to goat IgG (ab97100) horseradish peroxidase conjugated antibody, in a dilution 1∶5000. The bound antibodies were visualized by enhanced chemiluminiscence using ECL system (Amersham) and exposure to X-OMAT film. Signals were quantified by a densitometry by using Image Quant 5.2 (Molecular Dynamics) program.

### RNA isolation

Total RNA was isolated by TRIZOL reagent according to the procedure recommended by the manufacturer (Invitrogen, USA). Briefly, the tissue was homogenized in 1 ml of TRIZOL, and the aqueous phase was collected after the addition of chloroform. RNA was precipitated with isopropyl alcohol and washed with 75% ethanol. The RNA pellet was dried and re-dissolved in diethylpyrocarbonate-water. RNA concentration and purity were determined by measurement of absorbance at 260∶280 nm. For control of degradation of RNA, samples (2.5 μg of total RNA) were analyzed by 1.2% agarose electrophoresis.

### Real time qPCR

TaqMan PCR assays were carried out using Assay-on-Demand Gene Expression Products (Applied Biosystems, USA) for *Fto* (Rn 01538187, Applied Biosystems, CA), monoamine oxidase A (MAO-A; Rn 01430959, Applied Biosystems, CA) and catechol-O-methyltransferase (COMT; Rn 00561037, Applied Biosystems, CA). The gene expression assays contained primers for amplification of the target gene and the TaqMan MGB (Minor Groove Binder) probe 6-FAM dye-labelled for the quantification. Reactions were performed in a 25-μL reaction mixture containing 1X TaqMan Universal Master Mix with AmpErase UNG, 1X Assay Mix (Applied Biosystems) and cDNA template (10 ng RNA converted to cDNA). PCR was carried out in the ABI Prism 7000 Sequence Detection System at 50°C for 2 min, 95°C for 10 min, followed by 40 cycles at 95°C for 15 s and 60°C for 1 min. The experimental threshold was calculated based on the mean baseline fluorescence signal from cycle 3 to 15 plus 10 standard deviations. Each sample was run in triplicate and the mean value of each Ct triplicate was used for further calculations. A reference, endogenous control, was included in each analysis to correct the differences in the inter-assay amplification efficiency and all transcripts were normalized to ß actin (Rn 01412977, Applied Biosystems, CA) expression. For quantification, validation experiments were performed to determine the relevant endogenous control for the target genes. We tested ß actin and demonstrated that the efficiency of amplification was approximately equal for the endogenous control gene and all target genes. Serial dilutions of cDNAs were prepared and amplified by realtime PCR using specific primers and fluorogenic probes for target and endogenous control genes. The reaction mixture for endogenous control gene amplification consisted of 1X TaqMan Universal Master Mix with AmpErase UNG (Applied Biosystems), 1X Assay (6-FAM dye-labeled MGB probes) and cDNA (10 ng RNA converted to cDNA). Quantification was done using the 2^−ΔΔC^t method according to Livak and Schmittgen [11]. The obtained results were analyzed by the RQ Study Add On software for 7000 v 1.1 SDS instrument (ABI Prism Sequence Detection System) with a confidence level of 95% (p<0.05). The final result is reported as fold change relative to the calibrator and normalized to ß actin using the equation: N_sample_  = 2^−ΔΔCt^.

### Statistical analysis

One-way ANOVA with Tukey's posterior multiple comparison tests were employed for comparison of the experimental groups. The values were expressed as mean ± S.E.M. values of six animals, the level of significance being set at p<0.01 or higher.

### Immunofluorescence

Following decapitation, the brains were fixed in 4% paraformaldehyde and cryoprotected in 30% sucrose. After freezing at −80°C, brains were cut on a cryostat in 30 μm-thick coronal slices. The slices were first rehydrated for 30 min in 0.01 M PBS and then incubated for 15 min in 0,1% glycine at room temperature. Blocking was performed for 40 min in a solution containing 5% Donkey serum, 0,02% Triton-X and 0,01 M PBS. The incubation with primary antibodies was carried out overnight at 4°C in a solution containing 1% Donkey serum, 0,2 Triton-X and 0,01 M PBS. Neuronal nuclei were labeled with mouse anti-NeuN 1∶80 (Millipore, MAB377), while the polyclonal goat anti-*Fto* (goat polyclonal to *Fto* ab77547) antibody was used for the determination of the enzyme intracellular distribution. After washing the slices out three times for 10 min in 0,01 M PBS, secondary antibodies were applied. Secondary antibodies were Alexa Fluor 555-conjugated donkey anti-mouse 1∶200 (Invitrogen, A11055) for visualization of NeuN staining, and Alexa Fluor 488-conjugated donkey anti goat 1∶200 (Invitrogen, A31570) for visualization of *Fto* staining. Additionally, nuclei of the cells were stained with 4,6-diamidino-2-phenylindole (DAPI, 1∶4000, Molecular Probes, Eugene, USA) for 10 min, washed and mounted onto microscopic slides using Mowiol medium (Sigma Aldrich). Images of brain sections were obtained on a confocal laser-scanning microscope (LSM 510, Carl Zeiss GmbH, Jena, Germany) equipped with Ar Multi-line (457, 478, 488, and 514 nm) and HeNe (543 nm) lasers. Plan-Neofluar 20/0.5, and Plan-Apochromat 63/1.4 Oil DIC objective were used for image acquisition. Following acquisition, images were processed using the Zeiss LSM 510 Basic software package version 3.2.

In order to determine the proportion of Fto-positive neurons within the examined regions, two experimenters (blind of the animal status) manually counted the overall number of neurons in two representative confocal images of examined regions. Then, the parameters of the Image J software (National Institute of Health, USA) were adjusted, so that the results from manual and automated analysis for the same images match each other. Those parameters were then used to determine the number of neurons in all confocal images. Finally the number of the neurons with cytoplasmic *Fto*-expression was counted manually within all of the images and the results were expressed as a percentage of the total number of neurons.

## Results

**Figure 1:**
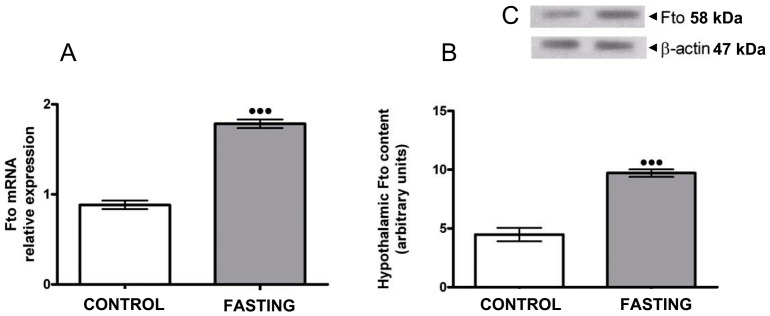
Hypothalamic *Fto* mRNA and protein levels were elevated after 48 hours of fasting. (A) RT-qPCR quantification of *Fto* transcript levels in the hypothalamus from control (n = 6) and fasted (n = 6) rats. *Fto* mRNA expression was significantly upregulated in the hypothalamus after 48 hours of fasting: ***p<0.001. Data represent means ± SEM. (B) Relative levels of *Fto* protein, normalised to β-actin, in the hypothalamus from control (n = 6) and fasted (n = 6) rats. *Fto* protein level was significantly increased in fasted rats in comparison to the controls: ***p<0.001. Data represents means ± SEM. (C) Representative Western blot of total proteins from control and fasted rat hypothalami probed for *Fto* (β-actin, loading control).

Hypothalamic gene expression study (Fig. 1A) showed that the relative expression of *Fto* mRNA was significantly upregulated in the animals which fasted for 48 h as compared to the controls (^***^p<0.001). Western blot analysis was used to show the hypothalamic protein expression pattern of *Fto* in given experimental groups (Fig. 1B). The immunoblot revealed approximately two-fold increase in *Fto* expression in fasting rats in comparison to that of controls (^***^p<0.001). These findings correlated with the *Fto* gene expression analyses.

**Figure 2:**
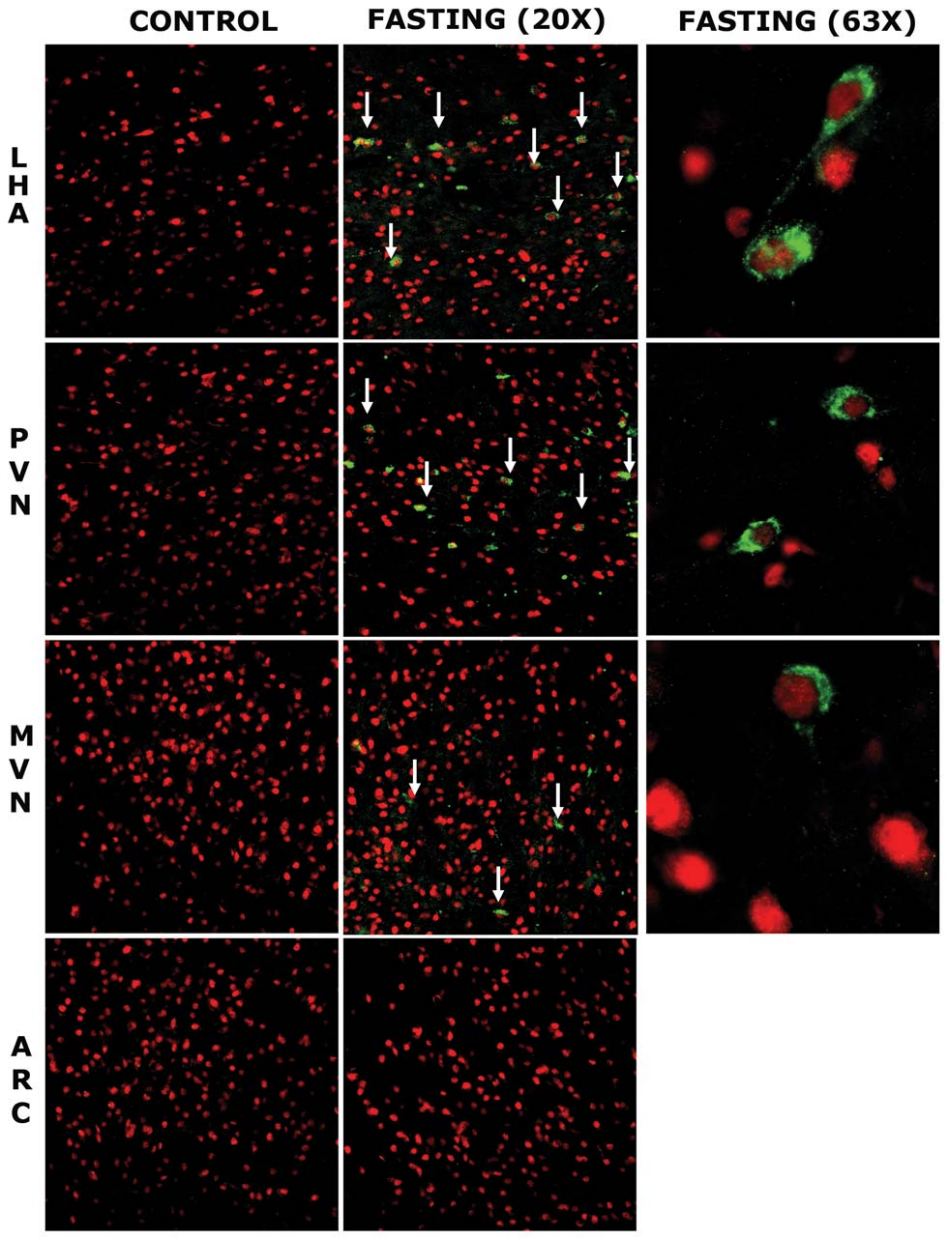
The effect of 48 h fasting on the *Fto* intracellular localisation in LHA, PVN, MVN and ARC neurons. Representative confocal images of sections from LHA, PVN, MVN and ARC of control (n = 3) and 48 h fasted (n = 3) rats. Sections were probed for *Fto* protein (green) and Neuronal Nuclear protein (NeuN, red). Cytoplasmic *Fto* localization was observed in some LHA, PVN and MVN neurons (marked whith white arrows), but not in ARC neurons of fasted rats. Cytoplasmic *Fto*-immunoreactivity was not observed in any of the examined hypothalamic regions in control rats.(LHA; lateral hypothalamic area, PVN; paraventricular nucleus, MVN; medioventral nucleus, ARC; arcuate nucleus).

**Figure 3:**
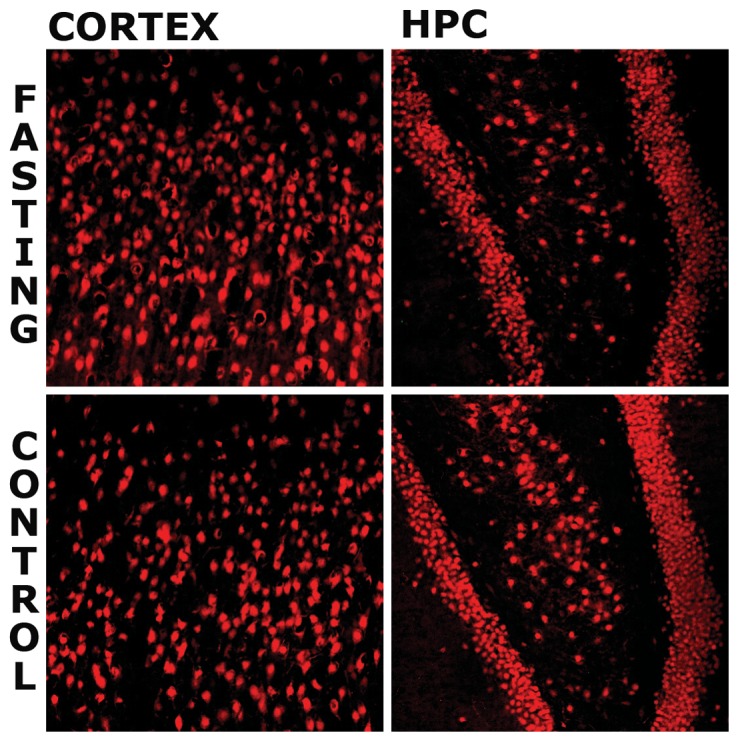
The effect of 48 h fasting on the *Fto* intracellular localisation in CORTEX and HPC. Representative confocal images of sections from CORTEX and ARC of control (n = 3) and 48 h fasted (n = 3) rats. Sections were probed for *Fto* protein (green) and Neuronal Nuclear protein (NeuN, red). h fasted (n = 3) rats. Sections were probed for *Fto* protein (green) and Neuronal Nuclear protein (NeuN, red). Cytoplasmic Fto-immunoreactivity was not observed in CORTEX and HPC neurons of either fasted or control rats. (HPC; hippocampus).

Double immunofluorescent staining was performed to confirm results from the Western blot. to address neuronal characterization of *Fto* (green fluorescence), using NeuN (red fluorescence) as a marker of neurons. DAPI was used to stain all the nuclei (blue). Figure 2. shows that 48 h food deprivation induced cytoplasmic *Fto* expression in some of LHA, PVN and MVN neurons. However, neither extranuclear *Fto*-immunoreactivity nor a change of intracellular localisation was observed in ARC neurons of either experimental group. Additionally, no change of the *Fto* cellular localisation was detected in the neurons of cortex and hippocampus (HPC) in either fasting or control animals (Fig. 3).

**Figure 4:**
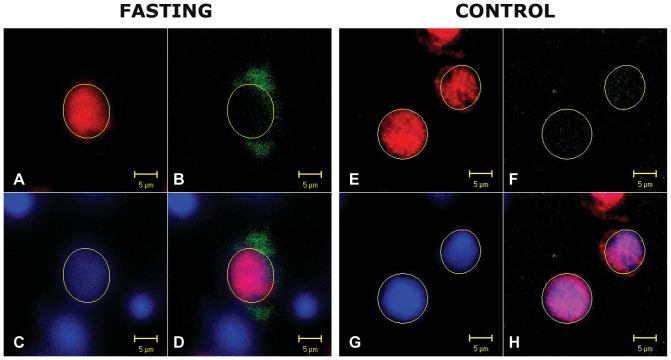
Intracellular *Fto* localisation in some of the hypothalamic neurons of 48 h-fasted and control rats. Representative confocal images of sections from control (n = 3) and 48 h fasted (n = 3) rats. Sections were probed for *Fto* protein (green) and NeuN (red) (4A, 4E). Note that the *Fto* antibody labeled cell nuclei of both food-deprived (4B) and control animals (4F), but the strongest *Fto* immunoreactivity was observed in the cytoplasm surrounding neuronal nuclei in LHA, PVN and MVN of the fasted rats (4B, 4D). DAPI was used to stain all nuclei (blue) (4C, 4G).

**Figure 5:**
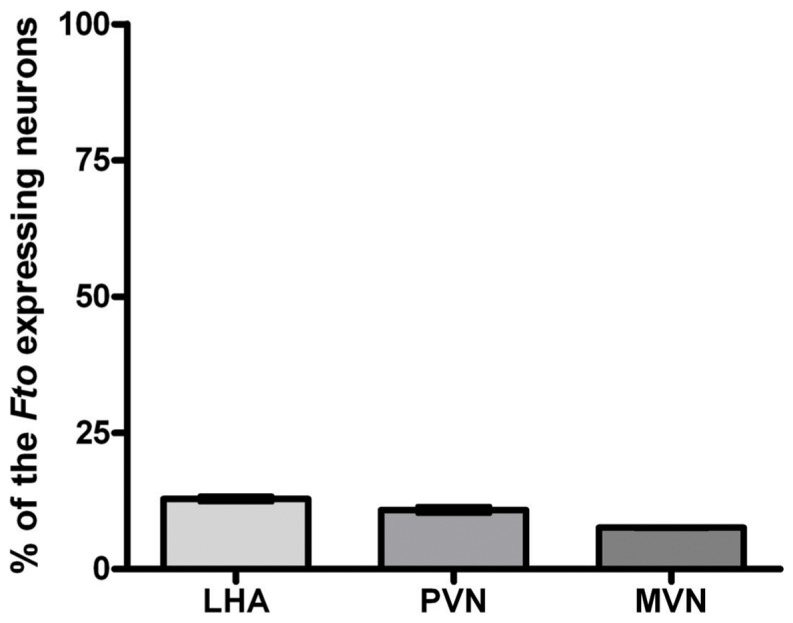
The proportion of neurons exhibiting cytoplasmic *Fto*-localisation in LHA, PVN and MVN of the rats subjected to 48 h fasting. (LHA; lateral hypothalamic area, PVN; paraventricular nucleus, MVN; medioventral nucleus).

Figure 4. depicts that some *Fto* immunoreactivity was detected in neuronal nuclei of both starved and control rats (4B, 4F). However, the strongest *Fto* immunoreactivity was observed in the cytoplasm surrounding neuronal nuclei in hypothalamic regions of the rats subjected to fasting (4B, 4D). The proportion of neurons that exhibited the change in *Fto* localisation was approximately 13% in LHA, 11% in PVN and 8% in MVN (Fig. 5).

**Figure 6:**
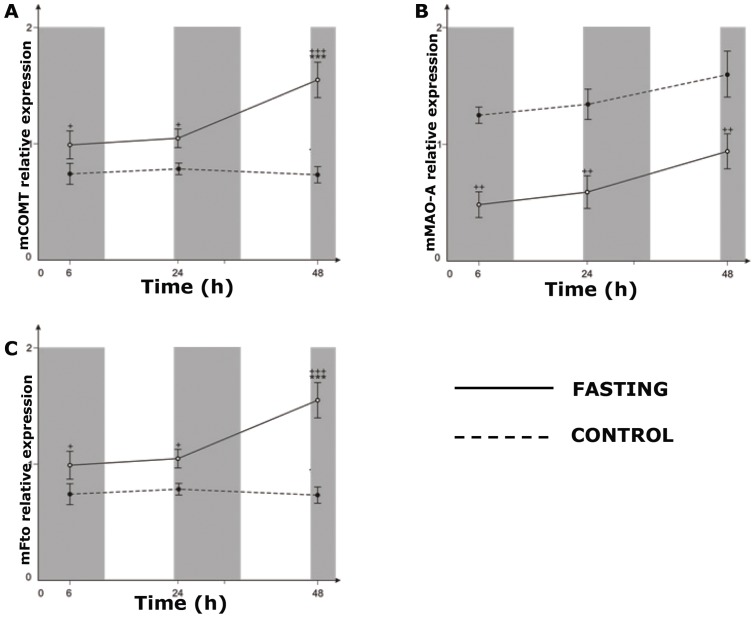
The effect of various fasting periods on relative expression of *Comt* mRNA, *Fto* mRNA and *Mao*-*A* mRNA in the hypothalamus. Gray areas indicate dark periods and white areas indicate light periods. Data shown are means ± S.E.M. Unlike *Mao-A* whose relative expression was downregulated by fasting throughout the examined period (6 h to 48 h)(^++^p<0.001), both *Fto* mRNA and *Comt* mRNA were upregulated in the identical time-dependent manner (^+^p<0.001, ^+++^p<0.001). (COMT; Catechol-O-methyltransferase, MAO-A; monoamine oxidse A).

Figure 6. shows that unlike monoamine oxidse A *(Mao-A)* mRNA whose relative expression was downregulated by fasting throughout the examined period (6 to 48 h) (^++^p<0.001), both *Fto* mRNA and catechol-O-methyltransferase (*Comt)* mRNA were upregulated by fasting in the identical time-dependent manner (^+^p<0.001, ^+++^p<0.001).

## Discussion

It is known that a change in nutritional status primarily affects *Fto* levels in hypothalamic regions (LHA, ARC, PVN, VMN) involved in the regulation of energy homeostasis [6]. Studies conducted on mice suggested that fasting induced a decrease in *Fto* mRNA in ARC. However, experiments on rats showed the opposite. In that respect, our findings are in line with those of Olszewski et al. [12], where the enhanced expression of both *Fto* mRNA and the protein was detected in hypothalamus of 48 h starved rats. Our immunofluorescence results have confirmed the increased *Fto* expression in some LHA, PVN and MVN neurons [3]. Surprisingly, although it had been unequivocally accepted that *Fto* is exclusively a nuclear protein [3], our studies revealed that the majority of *Fto* was localised in cytoplasm surrounding neuronal nuclei. Bearing in mind that we detected the upregulation of both the *Fto* mRNA and the protein expression as early as 6 hours after the food had been removed (Fig. 6C), it is unlikely that the *Fto* detected in cytosol was just newly synthesized protein not yet translocated into the cell nucleus. If this were the case, the increased amount of *Fto* would also have been detected within the nuclei of LHA, PVN and MVN neurons after 48 h of food deprivation. Despite the increase in the expression, the absence of food intake for 48 h did not result in the growth of the *Fto*-expressing neuronal subpopulations in the examined hypothalamic regions. Due to the weak nuclear Fto-immunoreactivity, we were only able to accurately determine the proportion of neurons displaying fasting-induced *Fto* extranuclear expression (approximately 13% in LHA, 11% in PVN and 8% in MVN).

Even though ARC was also documented to be involved in the regulation of appetite [6], neither an increase in *Fto* immunoreactivity nor a change in intracellular localisation of this enzyme was observed within the neurons of this hypothalamic area. Such findings are in agreement with previously published results that increased *Fto* expression in the rat ARC was associated with decreased food intake [13]. Taken together, these findings suggest that *Fto* expression is not universally regulated by food deprivation in all hypothalamic sites.

The question that clearly needs to be addressed is the one of the fasting-induced change of the *Fto* intracellular distribution in the LHA, PVN and MVN *Fto*-expressing neurons. Analysis of the *Fto* crystal structure has established that N-terminal domain (NTD) contains a nuclear localisation signal (NLS) [14]. It has previously been reported that masking of NLS can inhibit the nuclear import of proteins normally transported from cytoplasm to nucleus [15]. The abolishment of nuclear import can result from the cargo protein post-translational modifications, such as phosphorylation/dephosphorylation [16]. Namely, many NLSs are placed adjacently to phosphorylation sites and the state of phosphorylation at these sites can modulate the accessibility of NLS [16]. Bioinformatic analysis of rat *Fto* primary sequence (obtained from uniprot.org) has lead to the detection of numerous potential phosphorylation sites in ProSite database. One of them (PS00005|PKC_PHOSPHO_SITE (pattern) Protein kinase C phosphorylation site; 32–34: TpK) is in the vicinity of the N-terminally located NLS and although it is not yet functionally validated, it may be responsible for the observed change of the Fto intracellular localisation in the state of disturbed energy homeostasis.

Recent structural studies showed that single stranded RNA, rather than DNA, may be the prefered substrate of *Fto* [2]. If these findigs are viewed in the light of the cytosolic *Fto* localisation, one could hypothesise that rRNA may be one of the one substrates of the extranuclearly localised *Fto*. Namely, it was already confirmed that rRNA is most abundantly represented in total cell RNA [17]. Furthermore, it is known that 3-meU greatly contributes to the stability of rRNA and thus the stability of the entire ribosomal structure [18]. Demethylation of rRNA by *Fto* might be a mechanism which regulates the rRNA half-life. It has already been confirmed that fasting longer than 24 hours decreases the capacity for protein synthesis in the brain of an adult rat [19]. Taken together, these data could imply that the cytoplasmic *Fto* may destabilise ribosomes by demethylating rRNK and thus ultimately take part in decreasing the capacity for neuronal protein production in the state of prolonged food deprivation. Nontheless, given that the cytosolic *Fto* was not observed in the majority of neurons, it is unlikely that the decreased brain capacity for protein synthesis during prolonged fasting is mainly mediated by *Fto*. Certain additional mRNAs and transmitter systems that are specific for the neuronal subpopulations expressing *Fto* may also be affected by the enzyme in the state of disturbed energy homeostasis.

Fasting-induced cytosolic *Fto* accumulation may be important in regard to monoamine metabolism as well. It was reported that noradrenaline [20], serotonin and dopamine [21] all act within the hypothalamus to suppress food intake. Furthermore, the depletion of hypothalamic noradrenaline content (approx. 20%) was observed in 48 h food deprived rats [22]. The relevance of *Fto* activity in regard to monoamine metabolism was first recognized in the work of Tung et al. [23], where they found that increased *Fto* expression was associated with lowered expression of tyrosine hydroxylase (TH), an enzyme responsible for one of the steps in monoamine biosynthesis. Additionally, it was shown that *Fto* null mice had elevated catecholamine levels [24]. Our data indicate that, unlike *Mao A* whose hypothalamic expression was lowered (Fig. 6B), the expression of *Comt* was not only increased by fasting, but it went on to exhibit the identical pattern of expression as that of *Fto* from 6^th^ to 48^th^ hour of fasting (Fig. 6A, 6C). The comparison of fasting-induced changes of *Comt* and *Fto* expression indicate that methylation could be a dominant way of monoamine inactivation in the state of unfavorable energy balance. This assumption agrees with our previously stated hypothesis that the fasting-induced *Fto* presence in the cytoplasm may yield methyl groups which could further be used by *Comt* to inactivate monoamines.

In conclusion, our study demonstrates that fasting causes an increase in hypothalamic levels of both *Fto* mRNA and protein. Fasting did not affect *Fto* protein levels universally in all of the examined hypotalamic sites. Importantly, fasting has dramatically changed intracellular distribution of *Fto* in some LHA, PVN and MVN neurons, resulting in the majority of *Fto* being localized in the cytoplasm. We also hypothesize that increased methylation, as a result of increased *Fto* cytosolic expression, could represent a prominent way of monoamine inactivation in the state of food deprivation. Given the involvement of *Fto* in regulation of energy homeostasis, we believe that these findings could prove useful for future investigations in the field of energy homeostasis regulation.
